# Mirvetuximab Soravtansine Exposure and Incidence of Cataract Surgery

**DOI:** 10.1001/jamanetworkopen.2026.14557

**Published:** 2026-05-21

**Authors:** Jordyn F. Silverstein, Alexandra Smick, Edmund Tsui, Oladunni Alomaja, Bridget Johnson, Chi-Hong Tseng, Ritu Salani, Dennis J. Slamon, Dana M. Chase, Gottfried E. Konecny

**Affiliations:** 1 Division of Hematology/Oncology, Department of Medicine, University of California, Los Angeles; 2 Department of Obstetrics and Gynecology, David Geffen School of Medicine University of California, Los Angeles; 3 Department of Ophthalmology, Stein Eye Institute, David Geffen School of Medicine University of California, Los Angeles; 4 David Geffen School of Medicine at University of California, Los Angeles; 5 Department of Medicine Statistics Core, David Geffen School of Medicine at University of California, Los Angeles

## Abstract

This cohort study evaluates the incidence of cataract surgery among patients receiving mirvetuximab soravtansine to treat ovarian cancer.

## Introduction

Mirvetuximab soravtansine (MIRV), a folate-receptor alpha (FRα) targeted antibody-drug conjugate for the treatment of ovarian cancer (OC), has been associated with reversible corneal toxic effects including keratopathy, blurry vision, and dry eyes.^1^ While these short-term toxic effects are well described, data on the development of cataracts are limited.^2,3^ We describe the incidence of cataract surgery in patients treated with MIRV to better define long-term ocular safety.

## Methods

We conducted a matched cohort study in patients diagnosed with recurrent OC to examine the risk of cataract surgery after exposure to MIRV from January 2021 to December 2023. Patients with and without exposure to MIRV were matched by age at diagnosis. Multivariate logistic regression and Kaplan-Meier analyses were used. The institutional review board at the University of California, Los Angeles approved the study and granted a waiver of informed consent retrospective nature of the study, which posed minimal risk to participants. Reporting of this observational study adhered to the Strengthening the Reporting of Observational Studies in Epidemiology (STROBE) reporting guideline. All statistical analyses were performed using JMP Pro, version 16.0.0 (SAS Institute). Median time-to-event and corresponding 95% CIs were calculated. A second multivariate logistic regression was performed to assess the odds of cataract surgery among patients treated with MIRV only. All statistical tests were 2-sided, and a *P* value less than .05 was considered statistically significant. Further details on methods can be found in the eMethods in Supplement 1.

## Results

We identified 52 patients (mean [SD] age, 59.7 [11.1] years) exposed to MIRV with recurrent OC matched 1:1 for age to 52 nonexposed patients. There were no significant differences between groups in other established cataract risk factors, including hypertension, diabetes, smoking, and body mass index (Table 1). Nearly all patients exposed to MIRV (51 patients [98%]) were prescribed steroid eye drops. Systemic steroid exposure was uncommon; 46 (88%) received only steroid premedication. With a median follow-up after MIRV of 16.7 months and median (IQR) duration of MIRV treatment of 6.6 (3.4-12.2) months, cataract surgery was performed in 22 of 52 patients exposed to MIRV (42.3%) vs 6 of 52 in patients not exposed (11.5%) (adjusted odds ratio [aOR], 12.61; 95% CI, 2.95-53.81; *P* < .001) (Table 2).

**Table 1.** Baseline Demographic and Clinical Characteristics of Patients Exposed and Unexposed to Mirvetuximab Soravtansine

Characteristic	Patients, No. (%)		*P* value
	Exposed (n = 52)	Unexposed (n = 52)	
Age at diagnosis, y, mean (SD)	59.6 (11.4)	59.8 (11.1)	.92
Hypertension	14 (26.9)	23 (44.3)	.06
Diabetes	5 (9.6)	11 (21.2)	.09
Never smoker	37 (71)	28 (53)	.06
BMI at diagnosis, mean (SD)^a^	25.3 (5.3)	25.5 (6.1)	.89
Prior PARP inhibitor	32 (61)	34 (65)	.68
Prior bevacizumab	39 (75)	42 (80)	.48
Follow-up, y, mean (SD)	5.4 (3.0)	4.2 (3.1)	.02
Total lines of therapy, median (IQR)	5 (4-7)	3 (2-4)	<.001
Established with an ophthalmologist	50 (96)	29 (55.7)	<.001
Cataract before OC diagnosis	4 (7.7)	8 (15.3)	.21
Cataract surgery after diagnosis	22 (42.3)	6 (11.5)	<.001

Abbreviations: BMI, body mass index; OC, ovarian cancer; PARP, poly (ADC-ribose) polymerase.

^a^ Calculated as weight in kilograms divided by height in meters squared.

**Table 2.** Multivariate Logistic Regression Analysis to Estimate Odds of Cataract Surgery After Diagnosis

Variable^a^	OR (95% CI)	*P* value
MIRV exposure (reference: no)	12.61 (2.95-53.81)	<.001
Age	1.08 (1.02-1.16)	.01
BMI^b^	0.90 (0.78-1.01)	.08
Smoking (reference: former/current)	0.68 (0.19-2.32)	.53
Hypertension (reference: no)	2.32 (0.65-8.29)	.18
Diabetes (reference: no)	0.21 (0.03-1.63)	.10
Total lines of therapy	0.82 (0.59-1.10)	.18
Follow-up, y	1.32 (1.09-1.66)	.003

Abbreviations: MIRV, mirvetuximab soravtansine; BMI, body mass index; OR, odds ratio.

^a^ ORs for age, BMI, and other continuous variables represent the change in odds associated with a 1-unit increase in the variable.

^b^ Calculated as weight in kilograms divided by height in meters squared.

Among patients exposed to MIRV, adding 1 treatment cycle was associated with an aOR of 1.21 for cataract surgery (95% CI, 1.07-1.43; *P* = .01), and 19 patients (64%) treated for 6 or more months underwent cataract surgery. Patients who underwent cataract surgery had numerically higher rates of keratopathy (12 patients [60%] vs 7 [22%]), blurry vision (14 patients [70%] vs 7 [22%]), and dry eyes (13 patients [65%] vs 6 [19%]) compared with those who did not. The median (IQR) time from MIRV initiation to cataract surgery was 17.54 (95% CI, 14.29-27.79) months. Ten patients received cataract surgery while receiving MIRV treatment. None of these patients experienced complications from cataract surgery; however, 6 patients delayed reinitiation of MIRV treatment after surgery for a median (range) of 1 (1-4) week.

## Discussion

Despite the reversibility of MIRV-associated early ocular toxic effects, this cohort study found a high incidence of cataract surgery, suggesting the possibility of new delayed long-term ocular toxic effects. Among patients treated with MIRV for 6 or more months, nearly two-thirds required cataract surgery. Prior MIRV trials reported cataracts in 24% of patients (8% grade 3 or 4).^2,3^ The higher rate of cataract surgery observed in our study may reflect longer follow up allowing the capture of delayed onset toxic effects. In addition, the low rate of cataracts in published studies may be due to misattribution as an unrelated adverse event.^4^

The mechanism behind MIRV-associated cataracts remains uncertain since most MIRV ocular toxic effects affect the cornea, not the lens.^5^ However, FRα expression in the ciliary body and retina may cause intraocular inflammation and increase cataract risk.^6^ Corticosteroid eye drops, used prophylactically to mitigate keratopathy, may also play a role in cataract formation.^5^

Our study has the limitations of a retrospective single institution cohort study of small size including a potential risk of underreporting cataracts in the nonexposed group due to fewer eye examination. However, this should not have a major confounding effect on the results because the need for cataract surgery is primarily driven by patient-reported clinical symptoms and not an ocular examinations. Documentation of other ocular adverse events was incomplete due to ophthalmologic care received outside of our institution, limiting interpretation of these findings. Despite these limitations, our study underscores the need for further study of delayed long-term ocular toxic effects in patients treated with MIRV.

## Notes

Supplement 1. eMethods.

Supplement 2. Data Sharing Statement
