# Supplementary material for: Mirvetuximab Soravtansine Exposure and Incidence of Cataract Surgery
Source: JAMA Netw Open. 2026 May 21;9(5):e2614557. doi: 10.1001/jamanetworkopen.2026.14557 (PMC13195477; doi:10.1001/jamanetworkopen.2026.14557)
Supplement: Supplement 1. — eMethods. [file jamanetwopen-e2614557-s001.pdf]

# Supplemental Online Content

Silverstein JF, Smick A, Tsui E, et al. Mirvetuximab soravtansine exposure and incidence of cataract surgery. *JAMA Netw Open*. 2026;9(5):e2614557. doi:10.1001/jamanetworkopen.2026.14557

**eMethods.**

This supplemental material has been provided by the authors to give readers additional information about their work.

© 2026 Silverstein JF et al. *JAMA Network Open*.

**eMethods.**

*Patient Selection*

The University of California, Los Angeles (UCLA) Clinical and Translational Science Institute utilized the electronic health record clinical data mar to identify all patients treated with MIRV from January 2021 to December 2023 at UCLA. These patients were matched 1:1 by age at ovarian cancer diagnosis (± 5 years) to patients with ovarian cancer diagnosed prior to December 2023 who had not received MIRV. The Institutional Review Board approved the study and granted a waiver of informed consent. Data was collected by manual chart review and any uncertainty in the medical records pertaining to ovarian cancer factors or ophthalmology factors was discussed with an expert in gynecologic cancers (author GK), or an expert in ophthalmology (author ET), respectively. Reporting of this observational study adhered to the Strengthening the Reporting of Observational Studies in Epidemiology (STROBE) guidelines.

*Variables and Outcome Measures*

Collected variables included established cataract risk factors: age at diagnosis, history of hypertension, diabetes, and smoking (categorized as never, former or current), as well as body mass index (BMI) recorded closest to time of diagnosis. Patients were considered established with an ophthalmologist if there was an ophthalmology note in the medical record or reference to an ophthalmologist in an oncologist's note. Collected clinical variables included date of diagnosis, total lines of therapy, prior treatment with PARP inhibitors, bevacizumab, chemotherapy or other ADCs, total number of MIRV cycles, start and stop dates of MIRV, date of last follow-up, and vital status. The administration of steroid eye drops was collected via prescription records and date of prescription determined if they were prophylactic or treatment.

© 2026 Silverstein JF et al. *JAMA Network Open*.

The primary outcome was cataract surgery after ovarian cancer diagnosis, defined as a binary categorical variable based on ophthalmology notes or reported surgery dates in oncology records. The date of cataract surgery was the date of first cataract if there were two performed on the same patient for separate eyes. We also collected the type of cataract and other ocular toxicities as reported in ophthalmology notes. Patients who underwent cataract surgery prior to ovarian cancer diagnosis were included in the baseline data but excluded from the Kaplan Meier analysis. The date of data cutoff was May 2, 2025.

*Statistical Analysis*

All statistical analyses were performed using JMP Pro, Version 16.0.0 (SAS Institute Inc., Cary, NC). Descriptive statistics summarized baseline characteristics, and Chi-square or t-tests were used to compare MIRV-exposed patients with unexposed controls, as appropriate. Multivariate logistic regression analysis was used to determine the odds ratio of cataract surgery in the MIRV exposed and unexposed group. The model included variables that were either (1) established risk factors for cataract formation or (2) significantly different between groups, unless collinearity was present. Kaplan–Meier analysis was used to estimate time to cataract surgery among patients treated with MIRV, excluding those who underwent cataract surgery prior to MIRV. Median time-to-event and corresponding 95% confidence intervals were calculated using the Brookmeyer–Crowley method with log–log transformation. A second multivariate logistic regression was performed to assess the odds of cataract surgery among patients treated with MIRV only. All statistical tests were two-sided, and a p-value $<0.05$ was considered statistically significant.

© 2026 Silverstein JF et al. *JAMA Network Open*.
