# Supplementary material for: Mirvetuximab Soravtansine Exposure and Incidence of Cataract Surgery
Source: JAMA Netw Open. 2026 May 21;9(5):e2614557. doi: 10.1001/jamanetworkopen.2026.14557 (PMC13195477; doi:10.1001/jamanetworkopen.2026.14557)
Supplement: Supplement 2. — Data Sharing Statement [file jamanetwopen-e2614557-s002.pdf]

Data Sharing Statement

Silverstein. Mirvetuximab Soravtansine Exposure is Associated With an Increased Incidence of Cataract Surgery. *JAMA Netw Open*. Published May 21, 2026.
doi:10.1001/jamanetworkopen.2026.14557

Data
Data available: Yes
Data types: Deidentified participant data
How to access data: By request
When available: With publication

Supporting Documents
Document types: None

Additional Information
Who can access the data: researchers whose proposed use of the data has been approved
Types of analyses: for a specified purpose
Mechanisms of data availability: with investigator support
Any additional restrictions: under the discretion of the primary investigator
